# Has the Expected Shift in HIV-Related Cancers Occurred? Findings from a Long-Term HIV Cohort in Turkey

**DOI:** 10.3390/jcm15124818

**Published:** 2026-06-21

**Authors:** İnci Yılmaz Nakir, Melike Nur Özçelik, Rumeysa Gülistan Karaduman, Esra Zerdali

**Affiliations:** 1 Department of Infectious Diseases and Clinical Microbiology, Haseki Training and Research Hospital, İstanbul 34270, Türkiye; rgkaraduman@gmail.com (R.G.K.); esrayerlikaya@gmail.com (E.Z.); 2 Department of Infectious Diseases and Clinical Microbiology, Faculty of Medicine, Namik Kemal University, Tekirdag 59100, Türkiye; drmelikenozcelik@gmail.com

**Keywords:** HIV, AIDS-defining cancer, non-AIDS-defining cancer, late presentation, viral suppression, cancer epidemiology

## Abstract

**Background/Objectives**: Despite widespread antiretroviral therapy (ART) use, whether the expected transition from AIDS-defining to non-AIDS-defining cancers has occurred in settings with persistent late HIV presentation remains unclear. We examined long-term cancer patterns, determinants, and survival outcomes in a large HIV cohort. **Methods**: This retrospective, single-center cohort included 1419 people living with HIV followed between 2006 and 2024. Patients who developed malignancy were classified as AIDS-defining cancers (ADC) or non-AIDS-defining cancers (NADC). Immuno-virological parameters were assessed at HIV and cancer diagnosis. Survival was analyzed using Kaplan–Meier methods, and predictors of mortality were evaluated using Cox proportional hazards regression. Determinants of ADC development were assessed using multivariable logistic regression. Temporal changes were evaluated by trend analysis. **Results**: Sixty-six patients (4.6%) developed malignancy (31 ADC, 35 NADC). Late HIV presentation was common, with 72.7% having CD4+ T-lymphocyte counts < 350 cells/mm^3^ at cancer diagnosis, particularly among ADC cases. Most ADCs (93.5%) occurred within 24 months of HIV diagnosis. Overall survival did not differ between ADC and NADC groups (log-rank *p* = 0.14). Although mortality declined after 2015, temporal changes in ADC and NADC proportions did not reach statistical significance (*p* = 0.14). In Cox regression analysis, viral suppression before death or last follow-up was independently associated with lower mortality risk (HR 0.12; 95% CI 0.05–0.31). Lower CD4+ T-lymphocyte counts were associated with ADC development, and a CD4+ T-lymphocyte threshold of 295 cells/mm^3^ showed good discriminative performance (AUC = 0.83), although this cutoff should be interpreted cautiously due to the lack of external validation. **Conclusions**: In this long-term cohort from Türkiye, a clear epidemiological transition from ADC to NADC could not be demonstrated. The cancer spectrum remained strongly influenced by late HIV presentation and advanced immunodeficiency. Sustained viral suppression was independently associated with lower mortality risk, supporting the importance of early HIV diagnosis, timely ART initiation, and sustained virological control.

## 1. Introduction

The widespread use of combination antiretroviral therapy (ART) has markedly increased life expectancy among people living with HIV (PLHIV) and has substantially reduced AIDS-related morbidity and mortality. Globally, new HIV infections declined by approximately 40% between 2010 and 2024, while AIDS-related deaths decreased by 54% [1]. This significant improvement has changed clinical priorities in HIV care. The focus has shifted from an era dominated by opportunistic infections to one in which chronic comorbidities, particularly malignancies, have become increasingly prominent [2,3].

According to data from the Joint United Nations Programme on HIV/AIDS (UNAIDS), there were still approximately 1.3 million new HIV infections globally in 2024, with regional distributions showing significant variation. While a general decline is observed in the European Union/European Economic Area (EEA) region, Türkiye continues to have one of the fastest-growing HIV epidemics in the region [1,4].

Compared with the general population, PLHIV have a higher risk of developing cancer [5]. Since the early phase of the HIV/AIDS epidemic, certain malignancies—including Kaposi Sarcoma, specific subtypes of non-Hodgkin lymphoma (NHL), and cervical neoplasia—have been recognized as strongly associated with HIV-related immunosuppression [6]. Accordingly, since the 1990s, the Centers for Disease Control and Prevention (CDC) has classified these malignancies as AIDS-defining cancers (ADCs) [7].

However, although a clear epidemiological transition from ADC to NADC has been reported in high-income countries during the modern ART era, whether a similar transition has occurred in populations with persistently high rates of late HIV presentation remains uncertain [8,9,10]. Therefore, evaluating the spectrum, timing, and clinical outcomes of HIV-associated malignancies in settings with frequent late diagnosis remains important.

This study evaluates the epidemiological distribution and timing of malignancies in a large, long-term cohort of PLHIV between 2006 and 2024. It specifically examines the immunological profiles of these individuals at the time of cancer diagnosis. Furthermore, the study aims to assess mortality and survival outcomes, comparing ADC and NADC. These findings are expected to enhance the understanding of the HIV-related malignancy burden in populations with high new-diagnosis dynamics. Ultimately, this work aims to contribute to the strengthening of national clinical management strategies.

## 2. Materials and Methods

### 2.1. Study Design and Population

This single-center, retrospective descriptive study was conducted at the Infectious Diseases and Clinical Microbiology Clinic of Istanbul Haseki Training and Research Hospital. A total of 1419 PLHIV, who were followed between January 2006 and December 2024 and had complete clinical data, were evaluated. Among the cohort, individuals who developed a first primary malignancy during follow-up were evaluated in the malignancy analyses.

### 2.2. Participants and Eligibility Criteria

Individuals were included in the study if they were aged 18 years or older and had HIV infection confirmed by Western blot. At least one CD4+ T-lymphocyte count and one HIV RNA measurement at the time of HIV diagnosis were required. Malignancy diagnoses had to be confirmed using clinical, imaging, or histopathological methods. Individuals with a history of malignancy before HIV diagnosis were excluded. Patients with missing baseline laboratory data related to HIV diagnosis were not included in the study.

### 2.3. Data Collection

Demographic, clinical, and laboratory data were obtained from the electronic medical record system and the institutional database. We recorded variables including age, sex, HIV transmission route, sexual orientation, comorbidities, and antiretroviral therapy (ART) status. For each individual, CD4+ T-lymphocyte counts and HIV RNA levels were analyzed separately at two time points: at HIV diagnosis and at malignancy diagnosis. CD4+ T-lymphocyte counts were measured by flow cytometry (BD FACSLyric Analyzer, BD Biosciences, San Jose, CA, USA). HIV RNA levels were determined using the Cobas 6800 AmpliPrep HIV-1 Test system (Roche Diagnostics, Basel, Switzerland).

Mortality data were verified using the national electronic death notification system and hospital records. For all deceased individuals, the time from malignancy diagnosis to death was calculated in months.

### 2.4. Malignancy Classification and Definitions

Malignancy diagnoses were confirmed through clinical evaluation, imaging, and histopathological verification. All malignancies were then categorized using the International Classification of Diseases, 10th Revision (ICD-10). Detailed information on tumor type, histological features, stage, and treatment was extracted from medical records. According to the 1993 CDC classification, Kaposi sarcoma, non-Hodgkin lymphoma, and invasive cervical cancer were defined as ADC. All other malignancies were classified as NADC [7]. Finally, NADC subtypes were grouped descriptively by affected organ system and are presented in tables.

A CD4+ T-lymphocyte count of <350 cells/mm^3^ at the time of HIV diagnosis was defined as late presentation. A CD4+ T-lymphocyte cutoff of 200 cells/mm^3^ at the time of cancer diagnosis was additionally evaluated because it is widely used to define advanced immunodeficiency in HIV infection.

Viral suppression was defined as HIV RNA < 50 copies/mL. For survival analyses, viral suppression status was evaluated using the most recent HIV RNA measurement obtained before death or, for censored individuals, at the last available follow-up visit. Deaths in which malignancy was recorded on the death certificate as either the primary or a contributing cause were classified as “cancer-related deaths.” Survival time was defined as the period from the date of malignancy diagnosis to the date of death. Individuals who were alive at the end of follow-up were treated as censored in survival analyses.

Considering the international guideline updates recommending universal ART initiation regardless of CD4+ T-lymphocyte count, the increased access to ART in Türkiye, and the wider adoption of integrase inhibitor (INSTI)-based regimens over time, patients were descriptively evaluated in two separate periods according to the year of HIV diagnosis: before 2015 and after 2015.

### 2.5. Statistical Analysis

Statistical analyses were performed using IBM SPSS Statistics 29.0 and Python 3.10 software. Continuous variables were summarized as mean ± standard deviation or median (minimum–maximum), depending on their distribution characteristics. Categorical variables were expressed as frequencies and percentages. For group comparisons, the Chi-square test or Fisher’s exact test was used for categorical variables, where appropriate. For continuous variables, Student’s *t*-test or the Mann–Whitney U test was used, depending on the data distribution.

To evaluate independent determinants of ADC development, multivariable logistic regression analysis was performed. Variables considered clinically relevant based on prior literature and biological plausibility were evaluated for inclusion in the final model.

To assess predictors of mortality while accounting for follow-up time, Cox proportional hazards regression analysis was performed. Patients who were alive at the end of follow-up were treated as censored observations on 31 December 2024. Because of the limited number of mortality events, the multivariable Cox model was intentionally restricted to a small number of clinically relevant covariates to minimize the risk of overfitting and model instability.

To distinguish ADC from NADC, CD4+ T-lymphocyte counts at the time of diagnosis were assessed using ROC curves. The optimal threshold was determined using the Youden index. Survival analyses were performed using the Kaplan–Meier method, and differences between groups were compared using the log-rank test. To assess the temporal distribution of ADC and NADC by year of cancer diagnosis, malignancies were grouped into four periods (2006–2010, 2011–2015, 2016–2020, and 2021–2024). Linear trends in ADC and NADC proportions across periods were analyzed using the Cochran–Armitage trend test. A *p*-value of <0.05 was considered statistically significant in all analyses. All effect estimates are reported with 95% confidence intervals (CIs).

### 2.6. Ethics Approval and Consent to Participate

This study was approved by the Ethical Committee of the University of Health Sciences Haseki Training and Research Hospital, Istanbul, Türkiye (Approval No: 100-2024, dated 12 December 2024). All procedures were conducted in accordance with the required standards.

## 3. Results

### 3.1. Baseline Characteristics and Cancer Distribution

**Figure 1:**
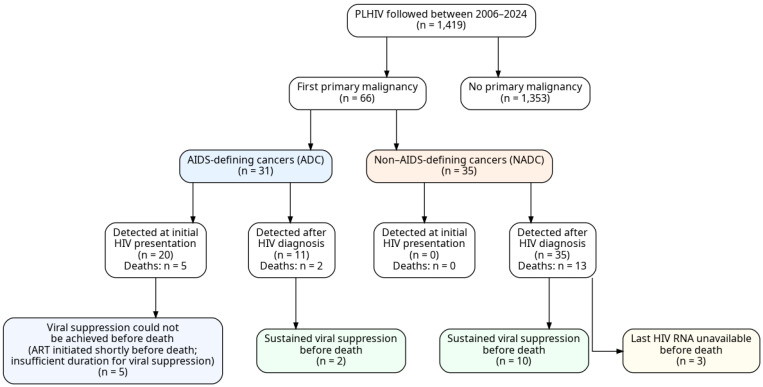
Flowchart of Patient Selection, Malignancy Classification, and Outcomes. PLHIV: people living with HIV; ADC: AIDS-defining cancer; NADC: non-AIDS-defining cancer.

Between January 2006 and December 2024, 1419 people living with HIV were followed. In this population, primary malignancy was identified in 66 individuals (4.6%). The detailed patient selection process and malignancy classification are presented in Figure 1.

Of these, 88.2% were male, and the mean age was 45.7 years. The mean CD4+ T-lymphocyte count at the time of HIV diagnosis was 302.6 cells/mm^3^. Of the 66 patients, 31 were classified as ADC and 35 as NADC. Among those diagnosed with malignancy, 83.3% were male, with a mean age of 45.0 ± 13.2 years. Patients diagnosed with ADC were younger than those in the NADC group. Age-stratified analyses demonstrated that ADC cases were more frequent among younger individuals, whereas NADC cases predominated in older age groups. Specifically, the proportion of patients aged > 45 years was substantially higher in the NADC group than in the ADC group (65.7% vs. 32.3%), while ADC cases were relatively more common among individuals aged ≤ 30 years (29.0% vs. 11.4%) (Table 1, *p* = 0.022).

CD4+ T-lymphocyte counts at the time of cancer diagnosis were significantly lower in the ADC group compared to the NADC group [150 (45–247) vs. 403 (250.5–522.5) cells/mm^3^, Mann–Whitney U test, *p* < 0.001]. Similarly, the proportion of patients with CD4+ counts ≤ 200 cells/mm^3^ at the time of cancer diagnosis was markedly higher in the ADC group than in the NADC group (71.0% vs. 34.3%; *p* = 0.004). Conversely, the rate of patients with CD4+ counts > 500 cells/mm^3^ was lower in the ADC group (3.2%) than in the NADC group (17.1%). No significant differences were found between the groups regarding other CD4+ subcategories. In the overall malignancy group, the rate of late presentation (CD4+ < 350 cells/mm^3^) was 72.7% (*n* = 48), and this rate was more pronounced in the ADC group. When comparing categorical variables between the two groups, no statistically significant differences were observed in terms of gender, sexual orientation, vital status, or HBV and HCV co-infections (Table 1, *p* > 0.05).

A total of 20 patients (30.4%) were diagnosed with cancer at their initial presentation with HIV infection, and all of these cases were in the ADC group. Among the seven individuals who died due to ADCs, only two (28.6%) had achieved sustained viral suppression before death. In the remaining five patients (71.4%), antiretroviral therapy had been initiated shortly before death, and an adequate duration of virological suppression could not be achieved. Among the 13 individuals who died due to NADCs, sustained viral suppression was present in 10 patients (76.9%). For the remaining three individuals, the last HIV RNA measurements prior to death were unavailable.

Among cancer types, Kaposi sarcoma (*n* = 18) was the most frequent malignancy in the ADC group, followed by non-Hodgkin lymphoma (*n* = 10). One patient had both Kaposi sarcoma and lymphoma simultaneously. In the NADC group, gastrointestinal system tumors were the most common malignancies (*n* = 10). Within this category, 50% were colorectal, 30% were upper gastrointestinal, and 20% were hepatopancreatobiliary in location. Respiratory system malignancies, consisting of lung and laryngeal cancers, were the second most common group *(n* = 7). Prostate cancer ranked third in frequency (*n* = 4). The detailed distribution of all subtypes is presented in Table 2.

### 3.2. Mortality and Survival Analysis

The overall mortality rate in the malignancy cohort was 30.3% (20/66). No significant difference in mortality was observed between the ADC and NADC groups (*p* = 0.199). In Cox proportional hazards analyses, increasing age was associated with a higher mortality risk in univariable analysis (HR 1.04; 95% CI 1.00–1.07; *p* = 0.031). No statistically significant association was observed between mortality and cancer type (ADC vs. NADC), CD4+ T-lymphocyte count ≤ 200 cells/mm^3^, or diagnosis period. In the multivariable Cox model, age showed a borderline association with mortality, whereas cancer type remained non-significant (Table 3). Given the limited number of mortality events, these findings should be interpreted cautiously.

**Figure 2:**
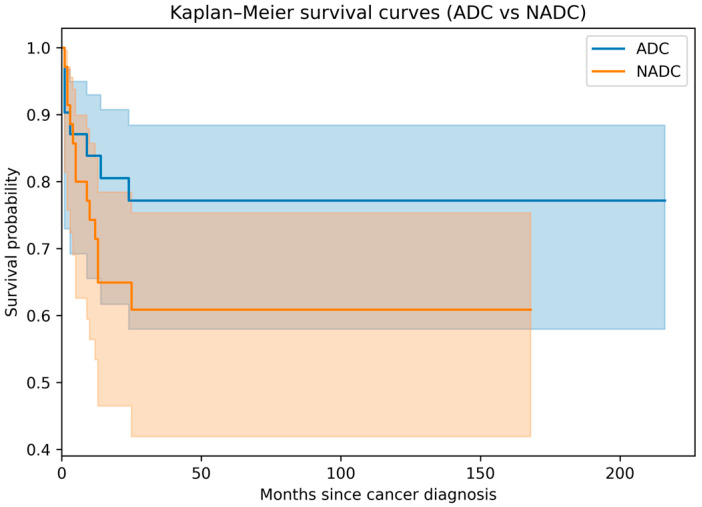
Kaplan–Meier survival curves comparing ADC and NADC groups. Kaplan–Meier survival curves showing overall survival probabilities for patients with AIDS-defining cancers (ADC) and non-AIDS-defining cancers (NADC). No statistically significant difference in survival was observed between the two groups (log-rank test, *p* = 0.14). Shaded areas represent 95% confidence intervals.

In the Kaplan–Meier survival analysis, there was no statistically significant difference in overall survival between the ADC and NADC groups (log-rank *p* > 0.05). Median overall survival was not reached in either group because fewer than 50% of patients experienced the event by the end of follow-up (Figure 2). The 12-month survival rates were 83.9% and 71.4% for the ADC and NADC groups, respectively. The 24-month survival rates were 77.4% for the ADC group and 64.6% for the NADC group. No statistically significant difference was observed between the survival curves (log-rank *p* = 0.14). Among cases resulting in death, the median time from cancer diagnosis to death was 3 months (range 1–24) in the ADC group and 5 months (range 1–25) in the NADC group.

### 3.3. Temporal Trends and Predictors

In time-dependent analyses, no significant difference was observed in the ADC/NADC distribution between the pre-2015 (*n* = 28) and post-2015 (*n* = 38) periods (*p* = 0.624). Age and CD4+ levels were also similar across these periods. In contrast, the mortality rate decreased significantly from 46.4% in the pre-2015 period to 18.4% in the post-2015 period (*p* = 0.018). Among deceased patients, the median time from cancer diagnosis to death was 7 months in the pre-2015 period and 5 months in the post-2015 period, with no statistically significant difference between the groups (Mann–Whitney U test, *p* = 0.905) (Table 4).

**Figure 3:**
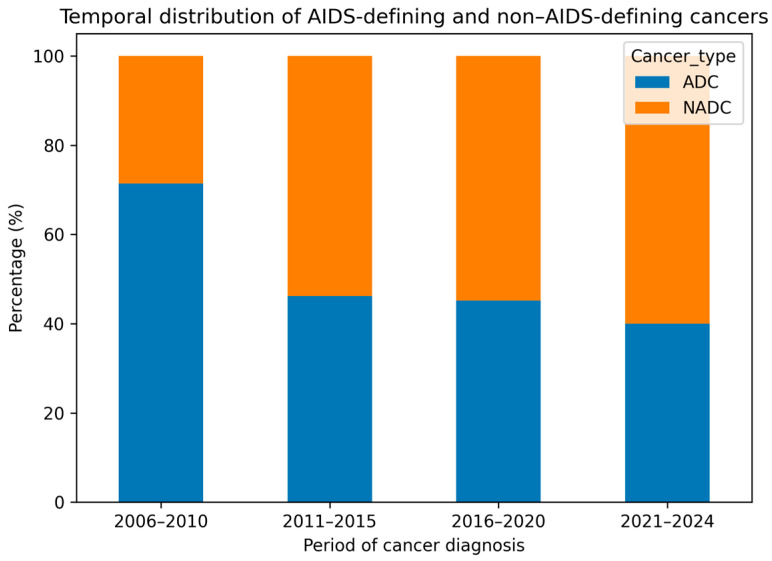
Temporal distribution of AIDS-defining and non-AIDS-defining cancers. Temporal distribution of AIDS-defining cancers (ADC) and non-AIDS-defining cancers (NADC) across four time periods (2006–2010, 2011–2015, 2016–2020, and 2021–2024). A decreasing trend in the proportion of ADC and a corresponding increase in NADC were observed over time; however, this trend did not reach statistical significance (Cochran–Armitage trend test, *p* = 0.14).

In the trend analysis based on year of cancer diagnosis, a decrease in the proportion of AIDS-defining cancers and an increase in the proportion of non-AIDS-defining cancers were observed over time. However, this change did not reach statistical significance (Cochran–Armitage trend test, *p* = 0.14). (Figure 3).

In the multivariable analysis, the CD4+ T-lymphocyte count at the time of malignancy diagnosis was identified as an independent predictor of ADC development (*p* = 0.004). This parameter also demonstrated the highest discriminative power (AUC = 0.83). The optimal cutoff value was determined to be 295 cells/mm^3^. In comparison, the AUC values for the CD4+ count at the time of HIV diagnosis and for age were 0.71 and 0.68, respectively.

The median duration between HIV diagnosis and malignancy diagnosis for all individuals was 24 months (IQR 0–84). This duration was 12 months (IQR 0–72) in the ADC group and 36 months (IQR 12–84) in the NADC group (*p* = 0.004). Within the first 24 months following HIV diagnosis, the rate of malignancy development was 93.5% in the ADC group, compared to 42.8% in the NADC group (Table 5).

## 4. Discussion

This study is one of the longest-running analyses in Turkey, covering a large cohort of PLHIV followed between 2006 and 2024. It evaluates the epidemiological characteristics, diagnostic timing, and survival predictors of malignancies in this population. Our findings show that an epidemiological shift from ADC toward NADC has not yet become prominent in our country. Although this shift has been widely reported in many high-income countries during the modern ART era, our data suggests a different trend. The primary reason for the persistent ADC burden appears to be the high rate of late presentation within our cohort. A significant majority of these patients are still diagnosed at a stage of advanced immunodeficiency. This situation aligns with recent reviews from low-, middle-, and upper-middle-income countries. These reports confirm that ADCs remain dominant in societies where late presentation remains a common challenge [8].

Many international studies have reported a significant decrease in ADCs, such as Kaposi sarcoma (KS) and non-Hodgkin lymphoma, during the post-modern ART era. An analysis in the United States involving over 847,000 PLHIV showed that the incidence of KS and diffuse large B-cell lymphoma has steadily declined since the 2000s. In contrast, the relative share of HPV-related malignancies and non-viral solid tumors has increased [9]. Similar trends were observed in the ICONA cohort. In that study, Kaposi sarcoma rates dropped from 39% to 19%, while the proportion of non-viral malignancies rose from 31% to 54% [10]. In our study, however, ADC and NADC rates did not show a statistically significant differentiation over the years. Although the temporal difference in ADC and NADC proportions did not reach statistical significance, the observed numerical increase in NADC cases may still suggest an evolving epidemiological trend that the present study may have been underpowered to detect.

This finding suggests that the epidemiological transition in our country has been delayed. At the time of HIV diagnosis, 72.7% of our patients had a CD4+ T-lymphocyte count of <350 cells/mm^3^. Furthermore, approximately one-third of the cases received their malignancy and HIV diagnoses simultaneously. These data are interpreted as a clinical reflection of the inadequacies in early testing and access to diagnosis. Aydın et al. reported similar findings in the ACTHIV-IST cohort [11]. Their data showed that most malignancies emerged either simultaneously with or shortly after the HIV diagnosis. They also noted that the majority of cases presented with CD4+ counts below 350 cells/mm^3^ [11].

The timing of malignancy diagnoses also supports this clinical picture. In our study, ADCs emerged as early as a median of 12 months. This finding is consistent with the classic pattern associated with advanced immunosuppression [12,13]. In contrast, NADCs were observed at a median of 36 months. This suggests a later risk profile associated with long-term follow-up, chronic inflammation, and aging. This difference in timing aligns with data reported in both Asian and European cohorts [9,14]. These results further demonstrate that late presentation continues to drive the ongoing burden of ADCs.

When examining the distribution of cancer types, KS stands out as a noteworthy feature in our country. A 2023 analysis in The Lancet Global Health identified Turkey as one of only two countries that did not experience a significant decrease in KS incidence [15]. Furthermore, that study highlighted that the increase in incidence was particularly prominent among males. In our study, KS cases were concentrated among males and did not show a marked decline over the years. This pattern is consistent with these broader epidemiological trends. International literature suggests that NADCs are influenced by regional risk profiles. Specifically, factors such as advanced age, heavy smoking, HBV/HCV co-infections, chronic inflammation, and lifestyle factors increase the prevalence of gastrointestinal and lung cancers [13,16,17,18]. In our cohort, gastrointestinal and respiratory system malignancies were the most frequently detected tumor groups among NADC subtypes. This finding aligns with both the data on PLHIV in Turkey and the international literature [11,13,16,17,18].

In our study, the CD4+ T-lymphocyte count was identified as the strongest predictor of ADC development. The high discriminative power obtained in the ROC analysis (AUC = 0.83) clearly demonstrates the direct relationship between ADC risk and immunodeficiency. Furthermore, the optimal cutoff value determined as 295 cells/mm^3^ reinforces this finding. These results are consistent with the RESPOND/D:A:D analyses. Those reports indicated that a CD4+ count of <350 cells/mm^3^ is an independent risk factor for both ADC and virus-related malignancies [19,20]. Similar findings have been reported in the Italian national cohort. In that study, cancer risk increased significantly at CD4+ levels below 200 cells/mm^3^. It was also noted that the 200–349 cells/mm^3^ range still carries a high risk [10].

In our study, no significant difference in mortality was observed between the ADC and NADC groups. In the Cox proportional hazards analysis, viral suppression before death or last follow-up was independently associated with lower mortality risk. These findings suggest that sustained virological control may play an important role in malignancy prognosis among PLHIV, regardless of cancer subtype. Although antiretroviral therapy had been initiated in most patients, detailed ART regimen data were not consistently available in this retrospective cohort. In several ADC cases resulting in death, ART had been initiated shortly before death, and sufficient time for durable virological suppression could not be achieved. In contrast, among NADC cases without viral suppression, irregular treatment use or inadequate virological response may have contributed to persistent viremia. These observations may partly explain the association between viral suppression and mortality observed in our cohort.

However, our mortality analysis has certain limitations. Data on tumor stage, metastatic status, and specific oncological treatment details were limited, preventing us from fully excluding residual confounders that might influence mortality. As reported in the literature, the survival difference between ADC and NADC vanishes in the presence of late presentation, low CD4+ counts, and detectable viral loads [21,22,23]. In the ACTHIV-IST cohort, NADC mortality was reported to be higher than ADC mortality [11]. In contrast, our study found similar mortality rates for both groups. This similarity may reflect the contribution of severe immunodeficiency and inadequate viral control to poor prognosis in the ADC group.

Time-dependent analyses revealed a significant improvement in survival over the years. The mortality rate decreased from 46.4% in the pre-2015 period to 18.4% in the post-2015 period. This improvement occurred despite age, CD4+ levels, and malignancy types remaining similar between the two eras. Although our study did not directly evaluate care processes during this period, several health system developments likely contributed to this positive trend. These include increased access to ART, faster initiation of treatment, and a more integrated structure for HIV–oncology care. These findings once again highlight the decisive role of early diagnosis, treatment continuity, and complete viral suppression on survival. These factors remain crucial in the management of malignancies in PLHIV, regardless of the specific tumor type.

Overall, our findings suggest that late presentation and inadequate viral suppression may substantially contribute to the HIV-associated malignancy burden in Türkiye. The high rate of ADC and the absence of a pronounced epidemiological shift may reflect that HIV is often diagnosed at advanced stages of immunodeficiency. These findings underscore the need to expand access to HIV testing and strengthen early diagnosis strategies at the national level. In survival analyses, viral suppression was independently associated with lower mortality risk. This finding suggests that timely initiation of ART and treatment continuity may play an important role in prognosis in both ADC and NADC management. In this context, improving the testing-to-diagnosis chain is essential. Furthermore, rapid ART initiation and sustained long-term virological suppression remain important strategies to reduce malignancy-related morbidity and mortality in people living with HIV.

This study has several limitations. The single-center and retrospective design may limit the generalizability of our findings. Due to the long follow-up period and heterogeneous tumor subtypes, certain key oncological variables—such as tumor stage, tumor grade, metastatic status, oncological treatment modalities, and performance status (Eastern Cooperative Oncology Group [ECOG])—could not be comprehensively included in the analyses. Consequently, residual confounding in the mortality analyses cannot be completely excluded. The limited sample size also restricted the statistical power of subgroup and temporal analyses, particularly within the non-AIDS-defining cancer subgroup. In addition, differences in follow-up duration and censoring patterns after cancer diagnosis may have influenced survival comparisons between ADC and NADC groups.

Furthermore, missing data on important lifestyle-related confounders, particularly smoking status and alcohol use, limited more comprehensive adjustment in the multivariable analyses. In addition, other oncogenic viral coinfections potentially associated with HIV-related malignancies, including Epstein–Barr virus (EBV), human herpesvirus-8 (HHV-8), and human T-lymphotropic virus type 1 (HTLV-1), were not systematically evaluated in this cohort. The temporal categorization used in this study was intended to reflect major changes in HIV management strategies in Türkiye over time; however, improvements in HIV care and ART access likely occurred progressively rather than at a single fixed time point.

Because this was a retrospective single-center cohort including only patients diagnosed and followed at our institution, selection bias cannot be excluded. In addition, referral patterns and local clinical practices may have influenced the observed malignancy spectrum and outcomes.

Despite these limitations, the long-term follow-up period and the availability of detailed immunovirological data represent important strengths of this study.

## 5. Conclusions

This long-term study suggests that the epidemiological shift from ADC to NADC, commonly observed in high-income countries, has not yet become clearly prominent in Türkiye. Persistently high rates of late HIV presentation and advanced immunodeficiency at diagnosis appear to remain major factors sustaining the burden of ADC. Although CD4+ T-lymphocyte count was identified as the strongest predictor of ADC development, sustained viral suppression was independently associated with lower mortality risk.

These findings underscore the importance of early HIV diagnosis, rapid initiation of ART, and sustained treatment continuity in reducing cancer-related morbidity and mortality. Moreover, the substantial contribution of NADCs to mortality highlights the need for strengthened oncological surveillance within routine HIV care. Expanding access to HIV testing and optimizing long-term virological suppression may represent important strategies to reduce the malignancy burden among people living with HIV in Türkiye. 

## Figures and Tables

**Table 1:** Characteristics of People Living with HIV/AIDS Diagnosed with Cancer.

	AIDS Defining Cancers (ADCs) (*n* = 31)	Non-AIDS Defining Cancers (NADCs) (*n* = 35)	*p*
**Age at HIV diagnosis** (years) Mean ± SD	40.55 ± 12.66	49.6 ± 12.71	**0.005** *
**CD4 count at HIV diagnosis (cells/mm^3^), median (IQR)**	113 (40–220)	279 (138–437)	***p* = 0.003** ^†^
**HIV RNA at HIV diagnosis (copies/mL), median (IQR)**	451,760 (171,902–1,365,725)	270,000 (68,314–1,100,188)	*p* = 0.197 ^†^
**CD4 count at cancer diagnosis (cells/mm^3^), median (IQR)**	150 (45–247)	403 (250.5–522.5)	***p* < 0.001**
**Sex**			0.521 ^#^
Female *n* (%)	4 (12.9)	7 (20)	
Male *n* (%)	27 (87.1)	28 (80)	
**Age groups at cancer diagnosis** (years)			**0.022** ^#^
≤30 *n* (%)	9 (29.0)	4 (11.4)	
31–45 *n* (%)	12 (38.7)	8 (22.9)	
>45 *n* (%)	10 (32.3)	23 (65.7)	
**Sexual orientation**			0.140 ^#^
Heterosexual *n* (%)	21 (67.7)	30 (85.7)	
MSM (Men who have sex with men) *n* (%)	10 (32.3)	5 (14.3)	
**CD4 count at cancer diagnosis** (cells/mm^3^)			**0.004** ^#^
≤200 *n* (%)	22 (71.0)	12 (34.3)	
>200 *n* (%)	9 (29.0)	23 (65.7)	
**HBV coinfection** *n* (%)	1 (3.2)	4 (11.4)	0.360 ^#^
**HCV coinfection** *n* (%)	0	0	
**Mortality** *n* (%)	7 (22.6)	13 (37.1)	0.284 ^#^

Abbreviations: ADC, AIDS-defining cancer; NADC, non–AIDS-defining cancer; HIV, human immunodeficiency virus; MSM, men who have sex with men; HBV, hepatitis B virus; HCV, hepatitis C virus; SD, standard deviation. Statistical analysis: Categorical variables were compared using the chi-square test with exact *p* values (^#^). ^†^ Mann–Whitney U test. Continuous variables were compared using Student’s *t*-test (*).

**Table 2:** Distribution of AIDS-defining and non-AIDS-defining malignancies among people living with HIV.

AIDS Defining Cancers (ADCs)	*n* (%)
Kaposi sarcoma	18 (58.1)
Non-Hodgkin lymphoma (NHL)	10 (32.3)
Kaposi + NHL	1(3.2)
Cervical cancer	2 (6.4)
**Total**	**31 (100)**
**Non AIDS Defining Cancers (NADCs)**	
Gastrointestinal system	10 (28.6)
Pulmonary	6 (17.1)
Central nervous system	6 (17.1)
Hematopoietic system	4 (11.4)
Prostate	4 (11.4)
Bone	2 (5.7)
Skin	1 (2.9)
Breast	2 (5.7)
**Total**	**35 (100)**

The data are presented as numbers (percentages). Abbreviations: ADC, AIDS-defining cancer; NADC, non–AIDS-defining cancer; NHL, non-Hodgkin lymphoma.

**Table 3:** Cox proportional hazards analyses of factors associated with mortality in patients with HIV-related malignancies.

Variable	Univariable HR (95% CI)	*p* Value	Multivariable HR (95% CI)	*p* Value
**Age (years)**	1.04 (1.00–1.07)	0.035	1.03 (1.00–1.07)	0.086
**ADC vs. NADC**	0.55 (0.22–1.39)	0.209	0.54 (0.20–1.45)	0.223
**CD4+ T-lymphocyte count ≤200 cells/mm^3^**	0.79 (0.31–1.98)	0.615	—	—
**Post-2015 cancer diagnosis**	0.47 (0.19–1.13)	0.092	—	—
**Viral suppression before death or last follow-up**	0.15 (0.06–0.36)	<0.001	0.12 (0.05–0.31)	<0.001

The data are presented as hazard ratios (HRs) with 95% confidence intervals (CIs). Patients alive at the end of follow-up were treated as censored observations on 31 December 2024. Because of the limited number of mortality events, the multivariable Cox regression model was intentionally restricted to a small number of clinically relevant covariates to minimize the risk of overfitting and model instability. Abbreviations: ADC, AIDS-defining cancer; NADC, non-AIDS-defining cancer; HR, hazard ratio; CI, confidence interval.

**Table 4:** Temporal comparison of cancer characteristics and outcomes according to HIV diagnosis era.

Variable	Pre-2015 HIV Diagnosis Period (*n* = 28)	Post-2015 HIV Diagnosis Period (*n* = 38)	*p* Value
**AIDS-defining cancer (ADC), *n* (%)**	13 (46.4)	18 (47.4)	0.624
**Non–AIDS-defining cancer (NADC), *n* (%)**	15 (53.6)	20 (52.6)	
**Age, years, median (IQR)**	42 (30–51.3)	47 (37–55.5)	0.203
**CD4+ T-cell count at cancer diagnosis (cells/mm^3^), median (IQR)**	285 (173.8–412.5)	236 (100–409)	0.429
**Mortality, *n* (%)**	13 (46.4)	7 (18.4)	0.018
Median time from cancer diagnosis to death among deceased patients, months *	7.0	5	0.905

The data are presented as median (interquartile range) or number (percentage), as appropriate. Temporal analyses were performed according to the year of HIV diagnosis. Comparisons between groups were conducted using the chi-square or Fisher’s exact test for categorical variables and the Mann–Whitney U test for continuous variables. * This value was calculated only among deceased patients and should not be interpreted as the median overall survival for the entire cohort. Abbreviations: ADC, AIDS-defining cancer; NADC, non-AIDS-defining cancer; IQR, interquartile range.

**Table 5:** Time interval between HIV diagnosis and malignancy diagnosis according to cancer type.

Variable	ADC (*n* = 31)	NADC (*n* = 35)	*p* Value
Time from HIV diagnosis to cancer diagnosis, months, median (IQR)	12 (0–72)	36 (12–84)	0.004
Cancer diagnosis within ≤24 months after HIV diagnosis, *n* (%)	29 (93.5)	15 (42.8)	<0.001

The values are presented as median (interquartile range) or number (%), as appropriate. Continuous variables were compared using the Mann–Whitney U test. Categorical variables were compared using the chi-square or Fisher’s exact test, as appropriate.

## Data Availability

The datasets generated and/or analyzed during the current study are not publicly available due to institutional and ethical restrictions related to patient confidentiality but are available from the corresponding author upon reasonable request.

## Abbreviations

The following abbreviations are used in this manuscript:

HIV	Human Immunodeficiency Virus
PLHIV	People Living with HIV
ART	Antiretroviral Therapy
ADC	AIDS-Defining Cancer
NADC	Non-AIDS-Defining Cancer
KS	Kaposi Sarcoma
NHL	Non-Hodgkin Lymphoma
CD4	Cluster of Differentiation 4
RNA	Ribonucleic Acid
HBV	Hepatitis B Virus
HCV	Hepatitis C Virus
MSM	Men Who Have Sex with Men
OR	Odds Ratio
CI	Confidence Interval
IQR	Interquartile Range
AUC	Area Under the Curve
HR	Hazard Ratio
